# Olaparib vs Cabazitaxel in Metastatic Castration-Resistant Prostate Cancer

**DOI:** 10.1001/jamanetworkopen.2021.10950

**Published:** 2021-05-24

**Authors:** Christopher J. D. Wallis, Zachary Klaassen, William C. Jackson, Robert T. Dess, Zachery R. Reichert, Yilun Sun, Daniel E. Spratt

**Affiliations:** 1 Department of Urology, Vanderbilt University Medical Center, Nashville, Tennessee; 2 Department of Surgery, Division of Urology, Medical College of Georgia at Augusta University, Augusta; 3 Georgia Cancer Center, Augusta University, Augusta; 4 Department of Radiation Oncology, University of Michigan, Ann Arbor; 5 Department of Medicine, University of Michigan, Ann Arbor; 6 Department of Biostatistics, University of Michigan, Ann Arbor; 7 Department of Radiation Oncology, University Hospitals, Case Western Reserve University, Cleveland, Ohio

## Abstract

This comparative effectiveness research compares survival end points and response rates among patients with metastatic castration-resistant prostate cancer (mCRPC) treated with olaparib and cabazitaxel using results from 2 phase 3 randomized clinical trials.

## Introduction

The PROfound trial^1,2^ led to US Food and Drug Administration (FDA) approval of olaparib for men with homologous recombination DNA repair (HRR)–deficient, metastatic castration-resistant prostate cancer (mCRPC) after progression on androgen signaling inhibitors (ARSis). Concerns regarding the broad FDA approval and the control group treatment have been raised. Switching ARSis from enzalutamide to abiraterone following progression (ie, ARSi switch), an approach commonly used in PROfound, has been reported to have PSA50 (prostate-specific antigen decline ≥50%) response rates as low as 1%.^3^ The CARD trial^4^ demonstrated an overall survival (OS) benefit with cabazitaxel vs ARSi switch in molecularly unselected patients with mCRPC who had received docetaxel and ARSi.^4^ This is a relatively similar patient population to that in the PROfound study, in which 65% of patients had received docetaxel and ARSi. Given the challenges of interpreting the findings of the PROfound study with available treatment options, we conducted a network analysis of the PROfound and CARD trials in patients with mCRPC who progressed on prior ARSi.

## Methods

Institutional review board approval was waived for this comparative effectiveness research study because it used previously published, publicly available data. This study followed the Preferred Reporting Items for Systematic Reviews and Meta-analyses (PRISMA) reporting guideline. A systematic review of published randomized clinical trials of cabazitaxel or poly(adenosine diphosphate–ribose) polymerase (PARP) inhibitor in patients with mCRPC following ARSi that reported OS and radiographic progression-free survival (rPFS) identified 2 relevant studies. We then compared olaparib (using the findings of the PROfound trial) vs cabazitaxel (using the findings of the CARD trial), using the common comparator (ARSi switch) (eFigure in the Supplement). We assessed OS, rPFS, objective response rate (ORR), and PSA50 response.^1,2,4^ We performed frequentist and bayesian network meta-analyses using the meta and rjags packages, respectively, in R version 3.4.2 (R Project for Statistical Computing). In frequentist analysis, we used generic inverse variance meta-analysis with fixed effects with a 2-sided α = .05 indicating statistical significance. In bayesian analyses, we determined the ranking probabilities of olaparib, cabazitaxel, and novel ARSi switch using the relative effect estimates.

## Results

**Figure 1.**
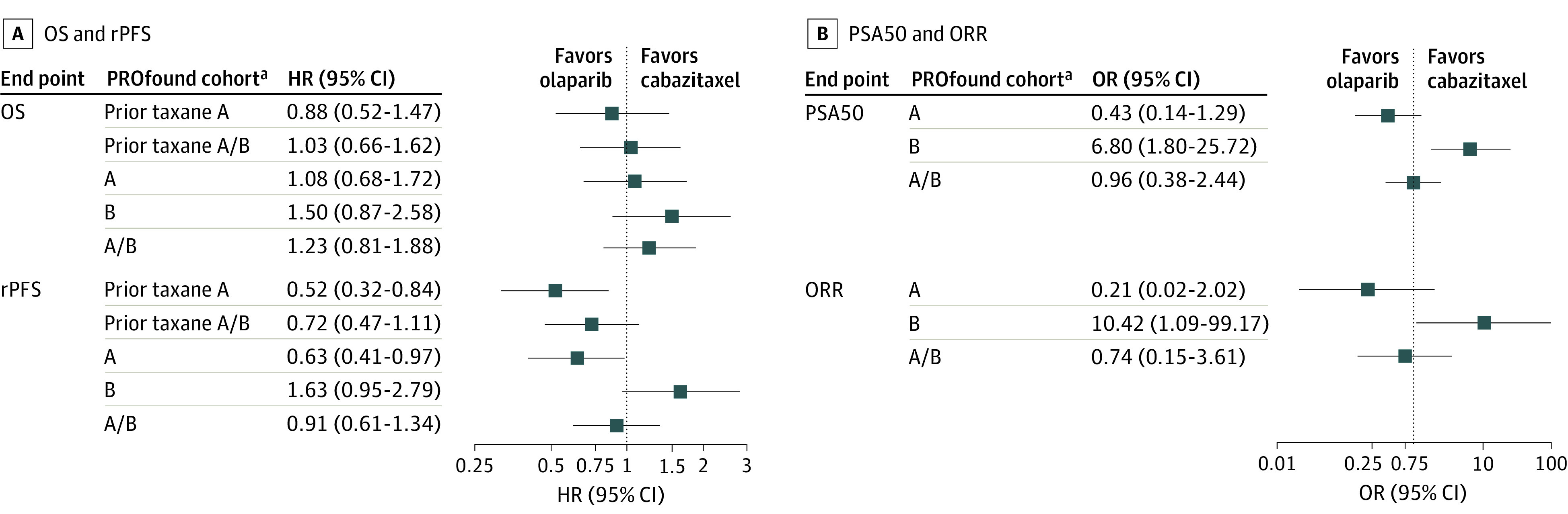
Frequentist Meta-analysis Assessing Survival End Points and Response Rates With Comparison of Olaparib and Cabazitaxel Across a Number of Prespecified Comparisons Using Different Cohorts From the PROfound Trial Tests for heterogeneous treatment effect between cohort A and cohort B revealed no significant difference for overall survival (OS; *P* for interaction = .37) but significant differences for radiographic progression-free survival (rPFS; *P* for interaction = .007), overall response rate (ORR; *P* = .02), and PSA50 (prostate-specific antigen decline ≥50%; *P* for interaction = .002). HR indicates hazard ratio; OR, odds ratio. Cohort A selected based on variants in *BRCA1, BRCA2,* and *ATM*. Cohort B selected based on variants in *BRIP1, BARD1, CDK12, CHEK1*, *CHEK2, FANCL, PALB2, PPP2R2A, RAD51B, RAD51C, RAD51D, RAD54L*. ^a^Patients in PROfound cohorts^1,2^ were compared with patients in the CARD trial.^4^

**Figure 2.**
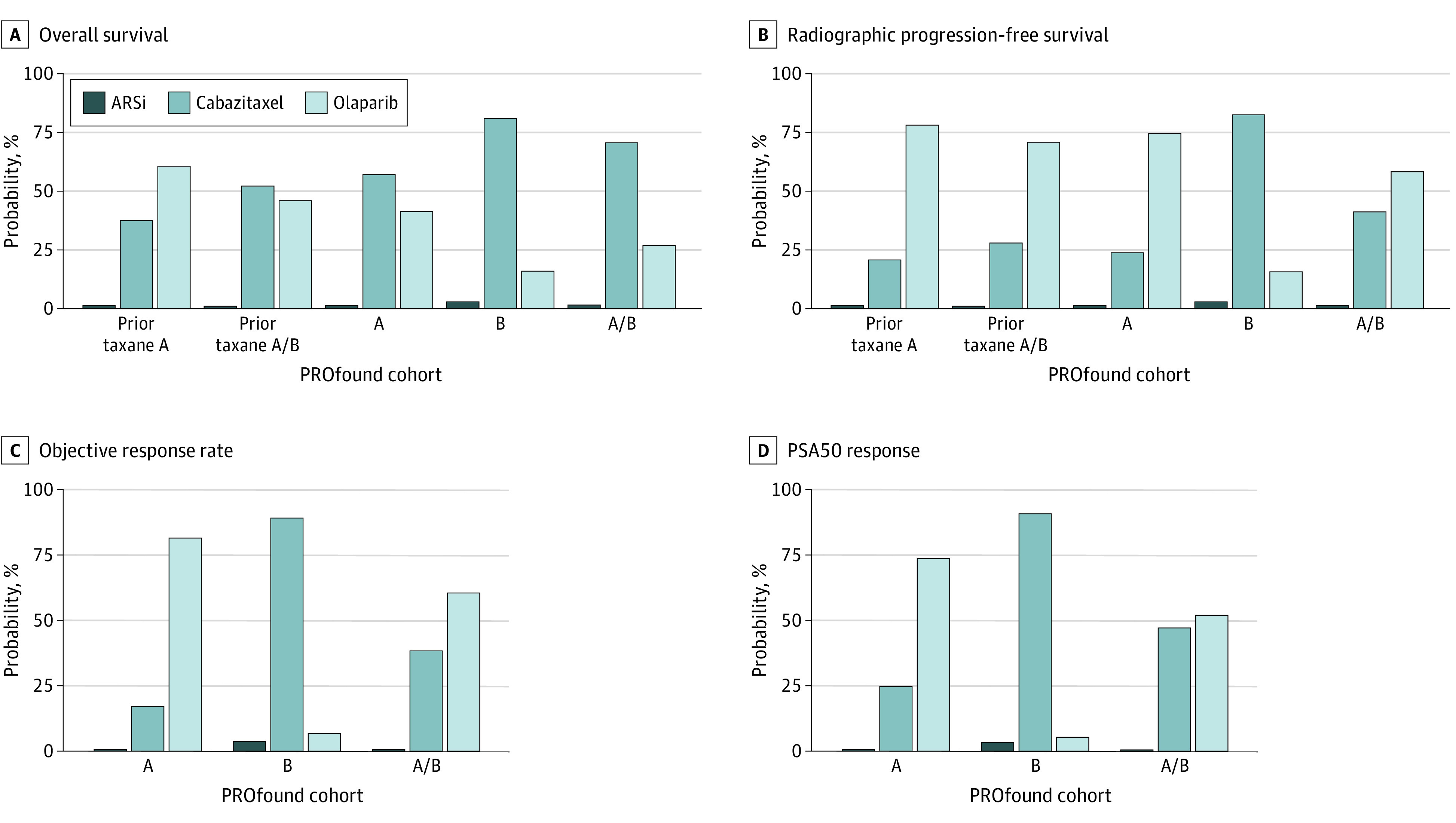
Rankograms From Bayesian Meta-analysis Assessing Overall Survival, Radiographic Progression-Free Survival, Objective Response Rates, and PSA50 Response Rankograms show which treatment that had the highest probability of having the best outcome, by cohorts reported in the PROfound trial. Cohort A selected based on variants in *BRCA1, BRCA2,* and *ATM*. Cohort B selected based on variants in *BRIP1, BARD1, CDK12, CHEK1*, *CHEK2, FANCL, PALB2, PPP2R2A, RAD51B, RAD51C, RAD51D, RAD54L*. ARSi indicates androgen signaling inhibitor; PSA50, prostate-specific antigen decline of at least 50%.

PROfound enrolled 387 patients with mCRPC who had progressed on prior ARSi and had variants in 1 of 15 HRR genes. CARD enrolled 255 men with mCRPC who had progressed on prior docetaxel and ARSi. Baseline characteristics were relatively similar between trials for median age (PROfound: 68 years [range, 47-91 years]; CARD: 70 years [range, 45-88 years]) and median PSA levels in the experimental groups (PROfound: 68 ng/mL [range, 24-294 ng/mL]; CARD: 62 ng/mL [range, 1.1-15 000 ng/mL]; to convert to micrograms per liter, multiply by 1.0). Among patients with *BRCA1*, (OMIM 113705); *BRCA2* (OMIM 600185), or *ATM* (OMIM 607585) variants (PROfound cohort A) and particularly among those who had received taxanes, olaparib was associated with superiod rPFS vs cabazitaxel (prior taxanes cohort A: hazard ratio, 0.52; 95% CI, 0.32-0.84; *P* = .007) (Figure 1A), with a 78% estimated probability of superiority (Figure 2B). In patients with 12 other HRR variants (PROfound cohort B) there was an 83% probability that cabazitaxel resulted in superior rPFS than olaparib (Figure 2B). OS was not significantly different between olaparib and cabazitaxel across any tested subgroup (Figure 1A), although bayesian analyses demonstrated a 60% to 80% probability of cabazitaxel being superior to olaparib in those who had not received prior taxanes (Figure 2A). Assessment of ORR and PSA50 similarly favored cabazitaxel in cohort B and olaparib in cohort A (Figure 2C and 2D), although no comparison demonstrated the statistically significant superiority of olaparib (Figure 1B).

## Discussion

In this study, the benefit associated with olaparib was reduced, eliminated, or inferior in specific subgroups of patients when treatment outcomes were compared with a more active standard of care, ie, cabazitaxel. While treatment with olaparib was associated with superior rPFS in patients with *BRCA1/2* variants, those with other HRR variants may have worse outcomes with this approach, which should be reassessed by national guidelines. Numerous active studies (NCT02952534, NCT02975934, NCT02854436, and NCT03148795) will add to the data regarding the role of PARP inhibitors in mCRPC.

There are limitations to this analysis, including the indirect nature and transitivity due to differences in the inclusion criteria of the 2 trials, unknown differential response of cabazitaxel to molecular subgroups used in PROfound, wide confidence intervals due to limited patient numbers, and the effect of crossover allowed in both trials. However, these hypothesis-generating data suggest reconsideration of the role of olaparib, and for which patients, in the mCRPC treatment armamentarium.

## Notes

Supplement. **eFigure.** Network Structure
