# Supplementary material for: Olaparib vs Cabazitaxel in Metastatic Castration-Resistant Prostate Cancer
Source: JAMA Netw Open. 2021 May 24;4(5):e2110950. doi: 10.1001/jamanetworkopen.2021.10950 (PMC8144926; doi:10.1001/jamanetworkopen.2021.10950)
Supplement: Supplement. — eFigure. Network Structure [file jamanetwopen-e2110950-s001.pdf]

Supplemental Online Content

Wallis CJD, Klaassen Z, Jackson WC, et al. Olaparib vs cabazitaxel in metastatic castration-resistant prostate cancer. *JAMA Netw Open*. 2021;4(5):e2110950. doi:10.1001/jamanetworkopen.2021.10950

eFigure. Network Structure

This supplemental material has been provided by the authors to give readers additional information about their work.

© 2021 Wallis CJD et al. *JAMA Network Open.*

eFigure. Network Structure

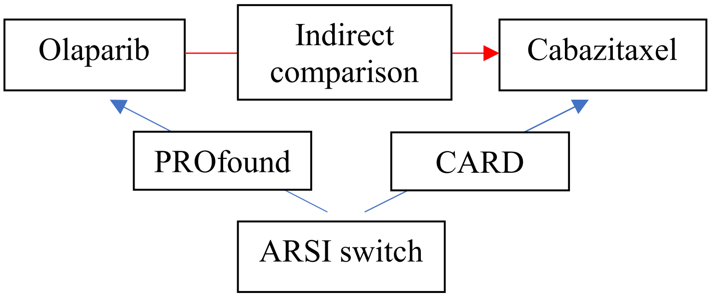

© 2021 Wallis CJD et al. *JAMA Network Open.*
